# Oleic Acid-Modified Oligochitosan Nanoparticles Preserve Bacteriophage Lytic Activity against Clinical *Escherichia coli* Isolates from Patients with Cystic Fibrosis

**DOI:** 10.1021/acsomega.6c04685

**Published:** 2026-09-08

**Authors:** Joselyn Aime García-Mar, Patricia Dolores Martínez-Flores, David Encinas-Basurto, Gerardo García-González, Gerardo E. Rodea-Montealegre, Refugio Pérez-González, Viviana Reyes-Márquez, Marco Antonio López-Mata, Natalia Hassan, Antonio Topete, Josué Juárez

**Affiliations:** † Departamento de Física, Facultad de Ciencias Exactas y Naturales, Universidad de Sonora, Hermosillo CP 83000, México; ‡ Departamento de Microbiología, Facultad de Medicina y Hospital Universitario “Dr. José Eleuterio González”, Universidad Autónoma de Nuevo León, Monterrey 64460, México; § Laboratorio de Investigación en Microbiología y Resistencia Bacteriana, Hospital Infantil de México, Federico Gómez, Ciudad de México 06720, Mexico; ∥ Departamento de Investigación en Polímeros y Materiales, Universidad de Sonora, Hermosillo CP 83000, México; ⊥ Departamento de Ciencias Químico-Biológicas, Facultad Interdisciplinaria de Ciencias Biológicas y de Salud, Universidad de Sonora, Hermosillo CP 83000, México; # Departamento de Ciencias de la Salud, Facultad Interdisciplinaria de Ciencias Biológicas y de Salud, Universidad de Sonora, Cajeme CP 85010, México; ∇ Programa Institucional de Fomento a la Investigación, Desarrollo e Innovación, Universidad Tecnológica Metropolitana, Santiago 8330383, Chile; ○ Departamento de Física de Partículas, Instituto de Materiales (iMATUS) e Instituto deInvestigación Sanitaria (IDIS), Universidad de Santiago de Compostela, Santiago de Compostela 15782, España

## Abstract

Cystic fibrosis (CF) lung infections are recurrent and frequently associated with multidrug-resistant bacteria, limiting the efficacy of the currently available antibiotic therapies. Bacteriophages have emerged as a promising alternative; however, their clinical translation is constrained by their limited stability and inefficient delivery within the airway microenvironment. Here, oleic acid-modified oligochitosan (OCS-OA) nanoparticles were developed as controlled-release nanocarriers to promote bacterial clearance during infection. Three OCS-OA formulations (10, 20, and 30% OA substitution) were modified via an amidation reaction and confirmed by attenuated total reflection Fourier transform infrared spectroscopy (ATR-FTIR). Surface tension analysis demonstrated the amphiphilic behavior and self-assembly of the modified oligosaccharides into micelle-like nanostructures. The bacteriophage (vB_Eco_K-02) was efficiently encapsulated (>90%), and the resulting formulations were characterized by scanning transmission electron microscopy (STEM), atomic force microscopy (AFM), dynamic light scattering (DLS), and ζ-potential. Formulations prepared with 10 and 20% OCS-OA preserved phage lytic activity against a clinical *Escherichia coli* strain isolated from CF patients, while the 30% formulation showed reduced activity, suggesting restricted phage release at a higher hydrophobic content.

## Graphical abstract

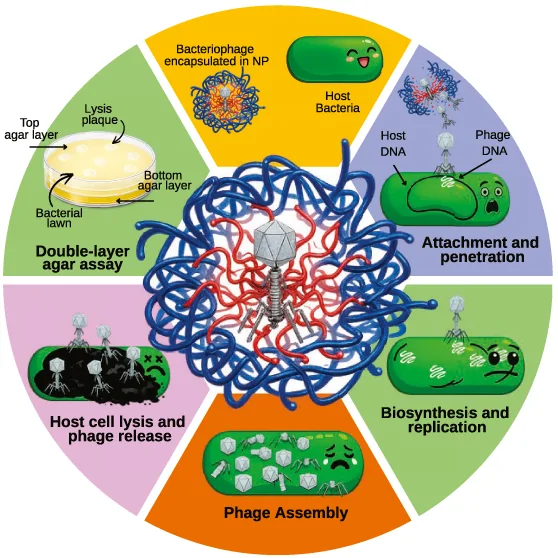

## Introduction

Cystic fibrosis (CF) is a chronic inherited disease that affects multiple organ systems. In the lungs, impaired mucociliary clearance leads to airway surface dysfunction and accumulation of abnormally thick, viscous mucus within the respiratory tract, making expectoration difficult and promoting mucus plugging.^1^ This retained mucus creates a permissive niche for bacterial colonization and infection, driving chronic airway inflammation and recurrent pulmonary exacerbations. Patients with CF are commonly treated with antibiotics (including aminoglycosides, β-lactams, and tetracyclines, among others).^2,3^ Although antibiotic therapy can be effective, airway infection frequently recurs because microbial populations shift under selective antibiotic pressure and are replaced by strains that are less susceptible or more resistant to the ongoing treatment. This process ultimately favors airway colonization and infection by major CF-associated pathogens, including *Staphylococcus aureus*, *Pseudomonas aeruginosa*, and the *Burkholderia cepacia* complex. These microorganisms are considered classical pathogens of progressive CF lung disease, as their increased abundance has been associated with accelerated lung function decline.^4^ However, increasing evidence indicates that nonclassical microorganisms, such as *Streptococcus* sp., *Prevotella* sp., and *Escherichia coli*,^5–8^ can also be detected in the airways of CF patients. In particular, *E. coli* has been isolated from sputum samples of CF patients, a reported prevalence of around 25%, suggesting that this bacterium can colonize the pulmonary airways and contribute to disease complexity. These MDR infections represent a major therapeutic challenge, as they reduce treatment efficacy and are associated with worse clinical outcomes and progressive health decline.^9^ Accordingly, there is a critical need to develop more effective and targeted antimicrobial strategies.

In this context, bacteriophages (viruses that infect and lyse bacteria) have re-emerged as promising therapeutic agents due to their host specificity and their ability to replicate locally at the site of infection.^10^ However, their clinical translation remains limited by several barriers, including reduced phage stability during storage and delivery, neutralization and clearance by the host immune system, and inefficient transport to and penetration within the infected airway microenvironment.^11^

Encapsulation of bacteriophages within nanoscale systems is a promising strategy to protect phages from premature inactivation and improve the localized pulmonary delivery. Successful formulation requires appropriate excipients such as disaccharides, amino acids, or polymer matrices. In this regard, oligochitosan (OCS)-based nanoparticles are attractive phage nanocarriers because they can shield phages from environmental stressors and physiological barriers. OCS is a chitin-derived natural biopolymer recognized for its biocompatibility, biodegradability, low toxicity, and ability to interact with cellular membranes and mucosal surfaces.^12,13^

Importantly, OCS can be chemically engineered owing to the presence of reactive functional groups, primarily hydroxyl (−OH) and primary amine (−NH_2_). The −NH_2_ group is commonly modified via an amidation reaction due to its straightforward and cost-effective chemistry.^14^ In particular, grafting fatty acids enables the modulation of the hydrophilic/hydrophobic balance, imparting an amphiphilic character to OCS and promoting its self-assembly into nanoscale structures capable of encapsulating diverse cargo, including both hydrophobic and hydrophilic molecules, as well as bacteriophages.

Here, oleic acid-modified OCS (OCS-OA) nanoparticles are presented as multifunctional bacteriophage nanocarriers and as an alternative therapeutic strategy for pulmonary bacterial infections in CF, with the goal of reducing or replacing antibiotic use and decreasing the recurrence of antibiotic-resistant infections. Three OCS-OA derivatives were synthesized with targeted OA substitution degrees (10, 20, and 30%) via an EDAC/NHS-mediated amidation reaction.^15^

## Materials and Methods

Oligochitosan (molecular weight: 4000–6000, >90% deacetylated deacetylated), hydrochloric acid, acetic acid, dimethylformamide (DMF), and 1-ethyl-3-(3-(dimethylamino)­propyl)-carbodiimide (EDAC) were purchased from Sigma-Aldrich. Deionized water (18.3 MΩ·cm) was used for all solution preparations.

### Derivatization and Purification of OCS-OA Conjugates

Three OCS-OA derivatives were synthesized by varying the degree of oleic acid (OA) substitution on the OCS backbone (10, 20, and 30%) via an amidation reaction. Briefly, OCS (0.5 g) was dissolved in 20 mL of 0.01 M HCl and stirred continuously for 24 h. In parallel, OA (0.166 g) was dissolved in 2 mL of DMF. Then, EDAC (0.118 g), previously dissolved in 1 mL of DMF, was added to the OA solution, and the mixture was stirred gently for 1 h to activate the carboxyl group. Subsequently, *N*-hydroxysuccinimide (NHS; 0.071 g), previously dissolved in 1 mL of DMF, was added, and the reaction mixture was maintained under constant stirring for 48 h. The resulting OCS-OA conjugates were purified by dialysis against deionized water using a dialysis tube membrane (MWCO 5000 Da) under gentle agitation to ensure uniform diffusion. The dialysate was replaced periodically to enhance the removal of residual reagents and solvents over 4 consecutive days.

### Surface Tension Measurements

Surface tension (γ) measurements were performed at different concentrations to determine the critical micelle concentration (CMC) of the OCS-OA derivatives. Measurements were carried out using a pendant drop tensiometer (Tracker Drop tensiometer, Teclis Instruments), equipped with a temperature-controlled chamber maintained at 25 °C. OCS-OA solutions (1 × 10^3^–1.18 × 10^6^) were prepared in deionized water, and 7.0 mL of each solution was transferred to a quartz cell. A stainless-steel needle connected to a glass syringe was used to generate a pendant droplet, and γ was continuously recorded as a function of time. Each run was stopped after 3600 s until γ reached a stable equilibrium value. All measurements were performed by triplicate, and results are reported as mean ± standard deviation (SD).

The CMC was determined from γ versus ln *C* plots as the intersection point between two linear fits corresponding to the semidilute adsorption region and the high-concentration plateau region. As previously described for polymeric surfactants, the γ-ln *C* profile typically exhibits three distinct regimes: (i) a dilute adsorption regime with minimal γ variation; (ii) a semidilute adsorption regime with a linear decrease in γ; and (iii) a high-concentration plateau indicative of surface saturation.^16^

The maximum surface excess concentration (Γ_max_) was calculated using the Gibbs adsorption isotherm

1 $$\Gamma_{\max}=-\frac{1}{\mathrm{nRT}}\left(\frac{d\gamma }{d\ln C}\right)$$

where *n* is the number of species formed upon surfactant dissociation in solution (*n* = 1 for the nonionic surfactant; *n* = 2 for the monovalent ionic surfactant), *R* is the universal constant gas constant, and *T* is the absolute temperature. The minimum area per molecule (*A*_min_) was calculated as

2 $$A_{\min}=\frac{1}{N_{A}\Gamma_{\max}}$$

where *N*_A_ is Avogadro’s number.

### Bacteriophage Encapsulation

Aqueous solutions (1% w/v) of each OCS-OA derivative (10, 20, and 30% substitution) were prepared in a final volume of 5 mL. Then, 200 μL of the diluted bacteriophage suspension (2:1 dilution from the concentrated stock of 3.4 × 10^9^ PFU/mL; phage vB_Eco_K-02) was added to each polymeric solution. The mixtures were subjected to ultrasonication at 10 kHz for 5 min while maintaining the suspension in an ice bath to prevent thermal degradation of the viral particles. The phage-loaded nanoparticles (ϕ-OCS-OA) were recovered by centrifugation at 13,000 rpm (17,000*g*) for 30 min at 15 °C. The resulting pellets were collected and resuspended in sterile water for subsequent characterization and functional assays.

### Assay for Quantifying Encapsulated Phages

The encapsulation efficiency (EE%) and phage viability were evaluated using the double-layer agar technique. Formulations prepared with 10, 20, and 30% OCS-OA-modified polymers were analyzed. 10-fold serial dilutions (1:10) of each encapsulated phage suspension were prepared. Agar medium was melted and maintained at 50 °C; subsequently, soft agar overlays were prepared by mixing 10 μL of bacterial host culture with 90 μL of each phage dilution. The mixtures were poured onto agar plates to form the top layer. After solidification and drying, the plates were incubated overnight at 37 °C. The following day, plaques were counted to determine PFU/mL and to assess lytic activity as a function of the polymer modification degree.

### Physicochemical Characterization

Physicochemical characterization of oleic acid-modified oligochitosan (OCS-OA) and bacteriophage-loaded OCS-OA (ϕ-OCS-OA) nanoparticles included particle size determination by dynamic light scattering (DLS) and surface charge measurement by ζ-potential analysis using a Zetasizer NanoZS90. Particle morphology and size distribution were evaluated by atomic force microscopy (AFM; SPM-4210, JEOL) and scanning transmission electron microscopy (STEM; JEOL-JSM-7800F) at the University of Sonora, as well as by STEM (ZEISS FESEM GEMINI-500) at the Universidad de Santiago de Compostela. Chemical modification of OCS was confirmed by attenuated total reflection Fourier transform infrared spectroscopy (ATR-FTIR). Colloidal stability of the nanoparticle suspension was monitored over 13 days by measuring hydrodynamic diameter and ζ-potential at predetermined time intervals.

### Statistical Analysis

All data are expressed as mean ± standard deviation (SD) from three independent measurements. Statistical analyses were performed using IBM SPSS Statistics (version 27). Hydrodynamic diameter and ζ-potential were analyzed by two-way ANOVA, with the OA substitution degree and storage time as fixed factors. One-way ANOVA followed by Tukey’s post hoc test was additionally performed at each storage time to compare formulations with different OA substitution degrees. Phage titers were transformed to log_10_(PFU/mL) prior to statistical analysis and compared among formulations by one-way ANOVA followed by Tukey’s post hoc test. Statistical significance was set at *p* < 0.05.

## Results and Discussion

Successful conjugation was confirmed by ATR-FTIR spectroscopy (Figure 1A). The absorption peaks at 1622 cm^–1^ and at 2920 and 2852 cm^–1^, assigned to the amide −C=O stretching and to the asymmetric/symmetric −CH_2_– stretching vibration of the OA alkyl chain, respectively, increased in intensity with the degree of substitution.

**Figure 1:**
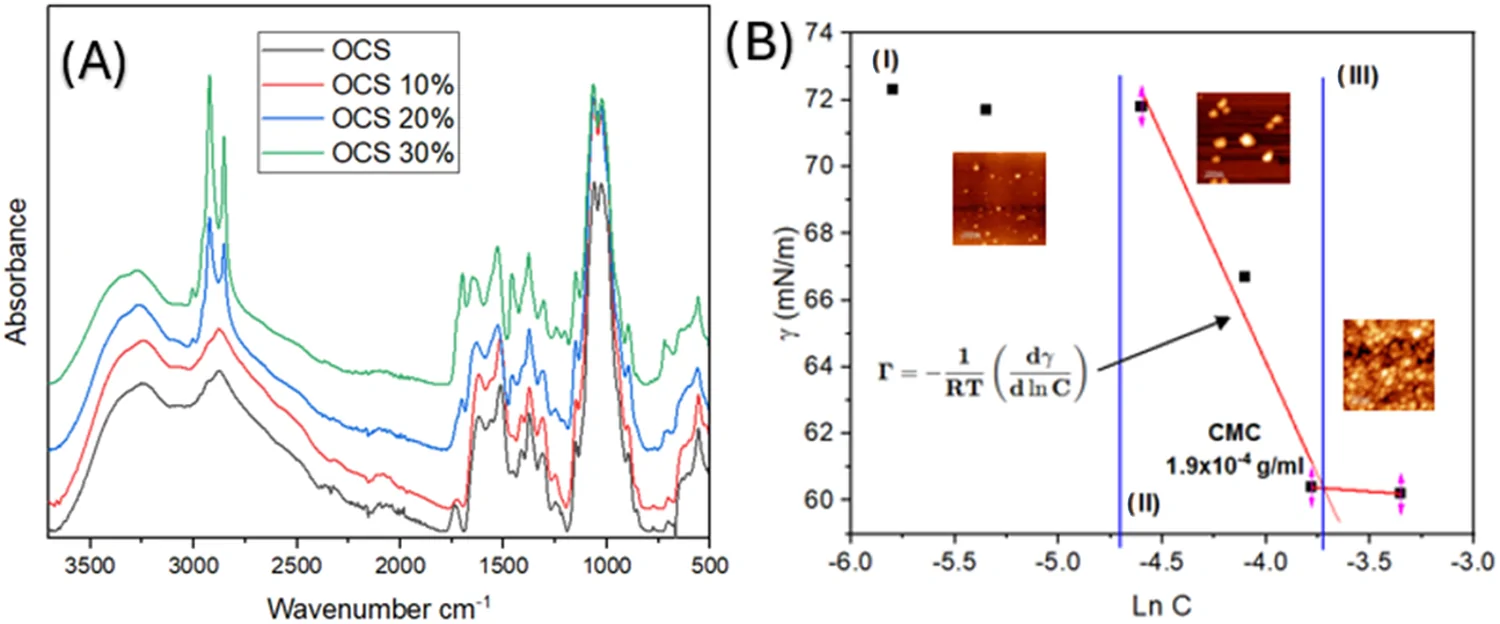
(A) ATR-FTIR spectrum of OQS purified by the dialysis method (pH 5), native OQS (black line), 10% OCS-OA (red line), 20% OCS-OA (blue line), 30% OCS-OA (green line). (B) γ as a function of ln *C* for OCS-OA (10% substitution).

The OCS-OA derivatives behaved as amphiphilic polymeric surfactants, where the hydrophilic glucosamine backbone and hydrophobic OA alkyl chains modulate the hydrophilic/hydrophobic balance, promoting self-assembly into micelle-like polymeric nanostructures. Key interfacial parameters, including the critical micellar concentration (CMC), maximum surface excess concentration (Γ_max_), and minimum area per molecule (*A*_min_), were determined from surface tension (γ) measurements (Figure 1B and Table S1). Figure 1B shows the characteristic γ versus ln *C* profile, exhibiting three distinct concentration regimes: (i) a dilute regime with minimal γ variation; (ii) semidilute regime in which γ decreases linearly with ln *C*; and (iii) a high-concentration plateau indicative of air–water interface saturation. Table S1 summarizes the CMC, Γ_max_, and *A*_min_ values. Notably, the CMC and *A*_min_ values decreased with increasing OA substitution, consistent with the progressively higher hydrophobic character of OCS-OA at higher grafting levels. The amphiphilic nature of OCS-OA enables spontaneous self-assembly into micelle-like structures in aqueous solution (Figure 1B, insets), where hydrophobic segments form the micellar core via water exclusion, while the hydrophilic glucosamine residues remain exposed at the micelle–water interface. The resulting OCS-OA nanoparticle exhibited a spherical morphology with an average size of ∼150 nm.

The capacity of OCS-OA nanoparticles to serve as a protective nanomatrix for bacteriophage loading and delivery was evaluated by encapsulating bacteriophages within the OCS-OA system (Figure 2). The bacteriophage used in this study (phage vB_Eco_K-02) was isolated from wastewater and kindly provided by Hospital Universitario “Dr. José Eleuterio González” (School of Medicine, UANL). vB_Eco_K-02 was genetically characterized in a previous report.^17^ Figure 2A,2B shows the STEM (50,000×; 100 nm scale bar) and AFM (3 μm × 3 μm; 600 nm scale bar) micrographs of vB_Eco_K-02, respectively. The phage exhibited a spherical morphology, with an average diameter of 62 ± 8 nm by STEM and 91 ± 15 nm by AFM. The observed morphology and size are consistent with the prior characterization of vB-Eco_K-02 by TEM, which reports an icosahedral morphology with a head of 52 nm length and a flexible tail of 75 nm.^17^

**Figure 2:**
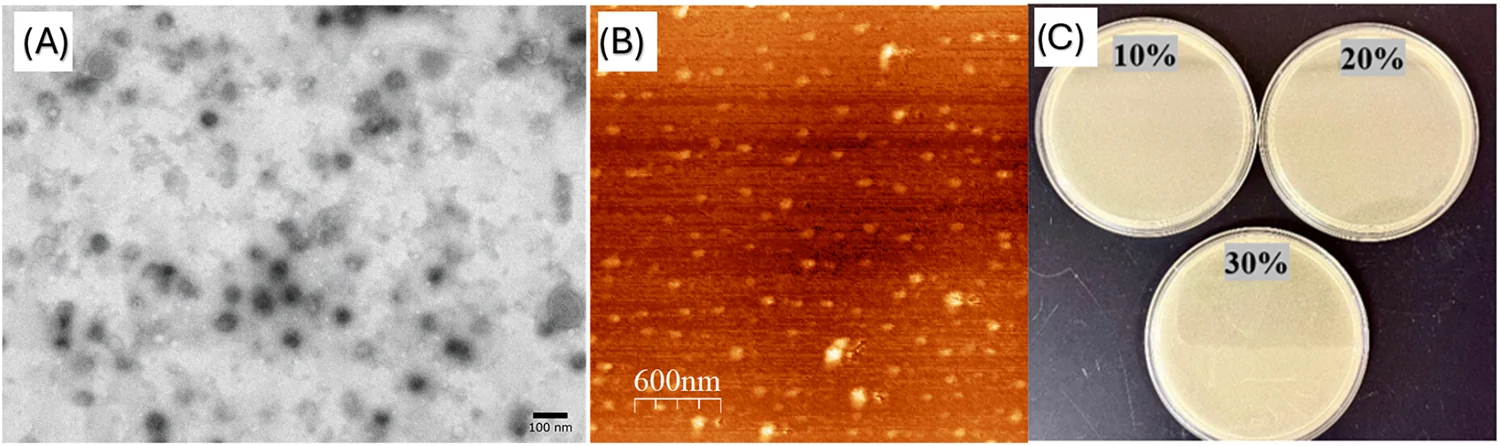
(A) STEM image of free vB_Eco_K-02 (scale bar: 100 nm). (B) AFM image of free vB_Eco_K-02 (scale bar: 600 nm). (C) Representative images of the double-layer agar assay plates used to quantify the nonencapsulated phage in the supernatants after the preparation of ϕ-OCS-OA nanoparticles with 10, 20, and 30% OA substitution.

Phage encapsulation was performed by adding vB_Eco_K-02 (3.4 × 10^9^ PFU/mL) to 5 mL of OCS-OA dispersion (1% w/v), followed by gentle sonication (10 kHz) for 5 min, maintaining the suspension in an ice bath. Phage-loaded OCS-OA nanoparticles (ϕ-OCS-OA) were recovered by centrifugation (13,000 rpm; 17,000*g*), and the supernatant was collected to quantify the nonencapsulated phage and determine the encapsulation efficiency (EE%). EE% was determined using the double-layer agar assay^17^ with a clinical *E. coli* strain isolated from CF patients and kindly provided by Centro Regional de Control de Enfermedades Infecciosas (School of Medicine, UANL, Monterrey, Mexico). All OCS-OA derivatives exhibited high encapsulation efficiencies (>90%; Figure 2C), consistent with large turbid zones and minimal to nondetectable translucent zones in the corresponding assay (Figure 2C).

Figure 3A,3B shows the representative morphology of ϕ-OCS-OA nanoparticles prepared from the 20% OA-substituted derivative. STEM imaging (Figure 3A) revealed nanoparticles with a core–shell-like architecture and an average diameter of 208 nm, consisting of a dense core surrounded by a diffuse outer corona. AFM imaging (Figure 3B) further supports this morphology, showing an average size of 214 nm. This structural arrangement suggests that bacteriophages are effectively incorporated into the OCS-OA matrix, forming the nanoparticle core, with the external OCS-OA for the corona-like nanoparticle, providing colloidal stability, consistent with DLS and ζ-potential measurements.

**Figure 3:**
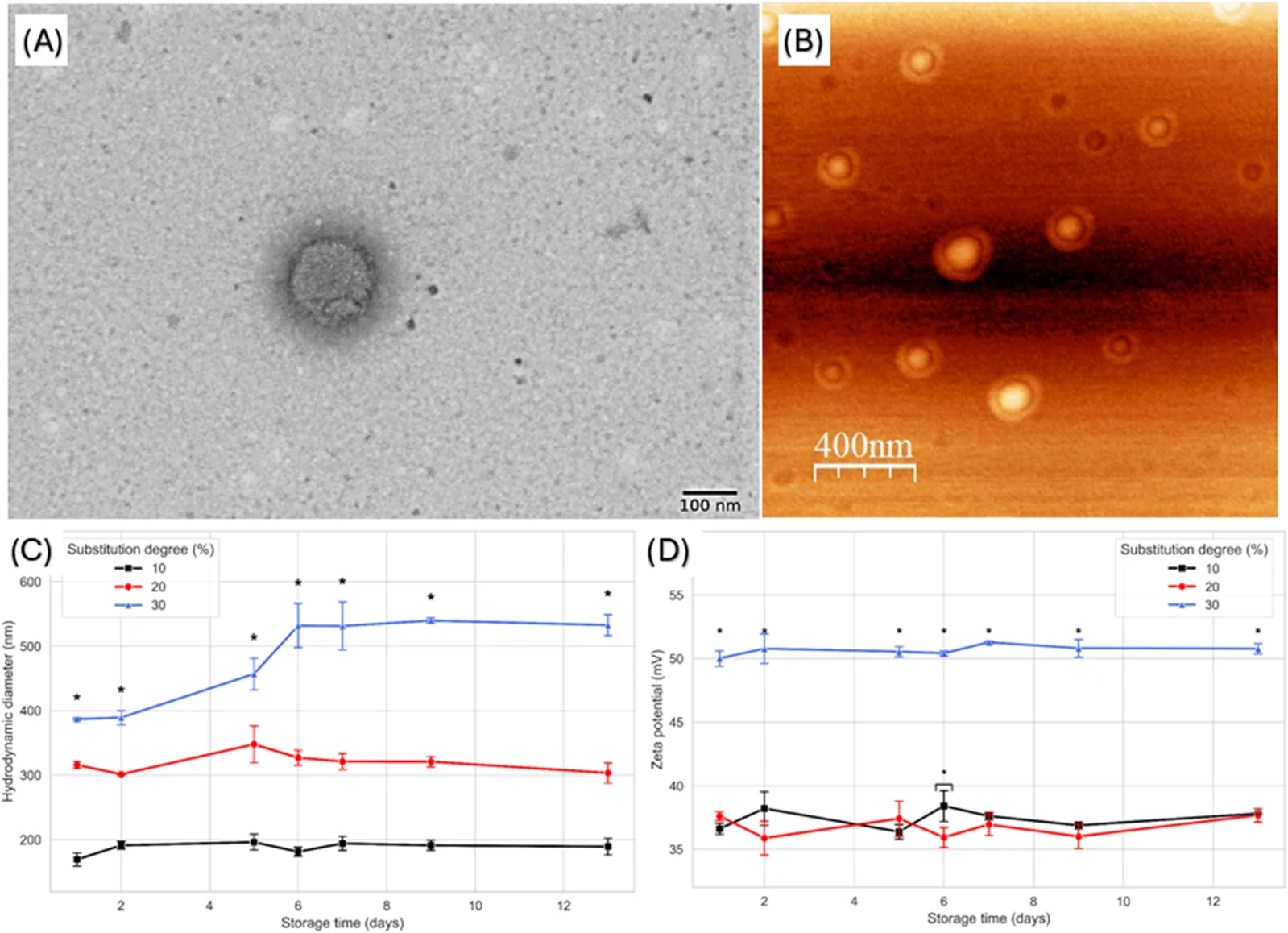
(A) STEM image of ϕ-OCS-OA 20% (scale bar: 100 nm). (B) AFM image of ϕ-OCS-OA 20%. (C) Hydrodynamic diameter stability of ϕ-OCS-OA nanoparticles with 10, 20, and 30% substitution degrees during storage. Data are expressed as mean ± standard deviation (*n* = 3). Asterisks indicate significant differences among substitution degrees at the same storage time according to Tukey’s post hoc test (*p* < 0.05). (D) ζ-Potential stability of ϕ-OCS-OA nanoparticles with 10, 20, and 30% substitution degrees during storage. Data are expressed as mean ± standard deviation (*n* = 3). Asterisks and brackets indicate significant differences among substitution degrees at the same storage time according to Tukey’s post hoc test (*p* < 0.05).

The colloidal behavior of ϕ-OCS-OA nanoparticles in aqueous media was strongly influenced by the degree of OA substitution (Figure 3C,D). The hydrodynamic diameter increased as the OA substitution degree increased, with initial values of 169.0 ± 10.0, 315.8 ± 5.5, and 386.7 ± 1.8 nm for the ϕ-OCS-OA formulations prepared with 10, 20, and 30% OA substitution, respectively (Figure 3C). Two-way ANOVA showed that the hydrodynamic diameter was significantly affected by the degree of substitution, storage time, and their interaction (*p* < 0.0001). Tukey’s post hoc test further confirmed significant differences among the three degrees of substitution at each storage time. Among the formulations, ϕ-OCS-OA 10% nanoparticles exhibited the smallest particle size and the highest dimensional stability, with only a modest increase from 169.0 ± 10.0 nm on day 1 to 189.0 ± 13.0 nm on day 13, reaching a maximum of 196.0 ± 12.0 nm on day 5. The 20% formulation exhibited intermediate hydrodynamic diameters, ranging from 315.8 ± 5.5 nm initially to 303.3 ± 15.6 nm on day 13, with a maximum of 347.7 ± 28.5 nm on day 5. In contrast, the 30% formulation showed the largest particle size and the most pronounced increase during storage, from 386.7 ± 1.8 nm at day 1 to 532.6 ± 16.6 nm at day 13, reaching a maximum of 539.6 ± 4.1 nm at day 9. This behavior suggests that increasing OA substitution promoted stronger hydrophobic interactions among OCS-OA chains, favoring the formation of larger nanoparticle assemblies.

ζ-Potential measurements indicated that all ϕ-OCS-OA formulations retained positive surface charges throughout the evaluated period (Figure 3D). At day 1, the ζ-potential values were 36.6 ± 0.4, 37.6 ± 0.3, and 50.0 ± 0.6 mV for the 10, 20, and 30% formulations, respectively. These values remained nearly constant after 13 days, reaching 37.8 ± 0.0, 37.7 ± 0.6, and 50.8 ± 0.4 mV, respectively. Two-way ANOVA showed that the ζ-potential was significantly affected by the substitution degree and by the interaction between the substitution degree and storage time (*p* < 0.05), whereas storage time alone did not have a significant main effect. Tukey’s post hoc test indicated that the 30% formulation consistently exhibited significantly higher ζ-potential values than the 10 and 20% formulations at all evaluated times, while the 10 and 20% formulations were generally comparable, except on day 6. The higher positive surface charge observed for the 30% formulation may be associated with a more compact or reorganized assembly of OCS-OA chains, which could favor the surface exposure of protonated amino groups. Overall, the positive ζ-potential values suggest that electrostatic repulsion contributed to colloidal stability; however, the marked increase in hydrodynamic diameter observed for the 30% formulation indicates that electrostatic stabilization was insufficient to fully counterbalance the stronger hydrophobic association promoted by the higher degree of OA substitution.

The lytic activity of ϕ-OCS-OA 10, 20, and 30% formulations was evaluated by the double-layer agar assay against a clinical *E. coli* strain isolated from patients with cystic fibrosis (Figure 4A). The phage-loaded formulations produced visible lysis plaques, confirming that the encapsulated phages retained infective capacity after formulation. Quantitative phage titers were further compared among formulations (Figure 5). The ϕ-OCS-OA 10% formulation exhibited the highest lytic activity, with a phage titer of 2.60 × 10^9^ ± 8.00 × 10^8^ PFU/mL, followed by the 20% formulation with 1.60 × 10^8^ ± 7.00 × 10^7^ PFU/mL. In contrast, the ϕ-OCS-OA 30% formulation showed markedly reduced lytic activity, with a titer of 4.50 × 10^5^ ± 6.00 × 10^4^ PFU/mL. One-way ANOVA performed on log_10_-transformed PFU/mL values showed significant differences among formulations with different OA substitution degrees (*p* < 0.0001), and Tukey’s post hoc test confirmed that all formulations differed significantly from each other.

**Figure 4:**
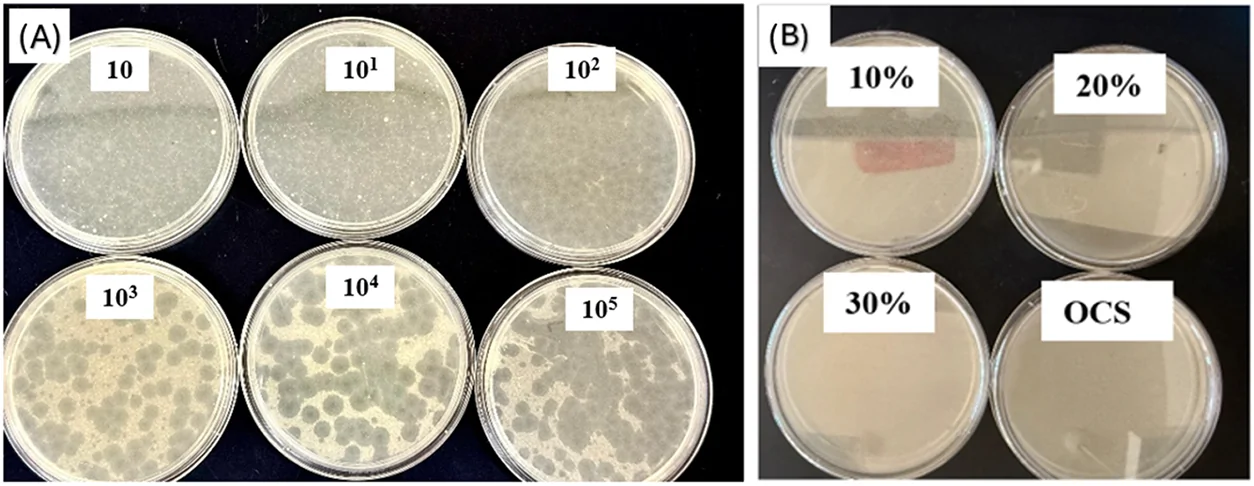
(A) Double-layer agar assay plates used to evaluate the lytic activity of phage encapsulated in ϕ-OCS-OA 10% across serial dilution (10, 10^2^, 10^3^, 10^4^, and 10^5^). (B) Double-layer agar assay plates showing no lytic activity for empty nanoparticles (OCS-OA 10, 20, and 30%) and unmodified OCS (phage-free controls).

**Figure 5:**
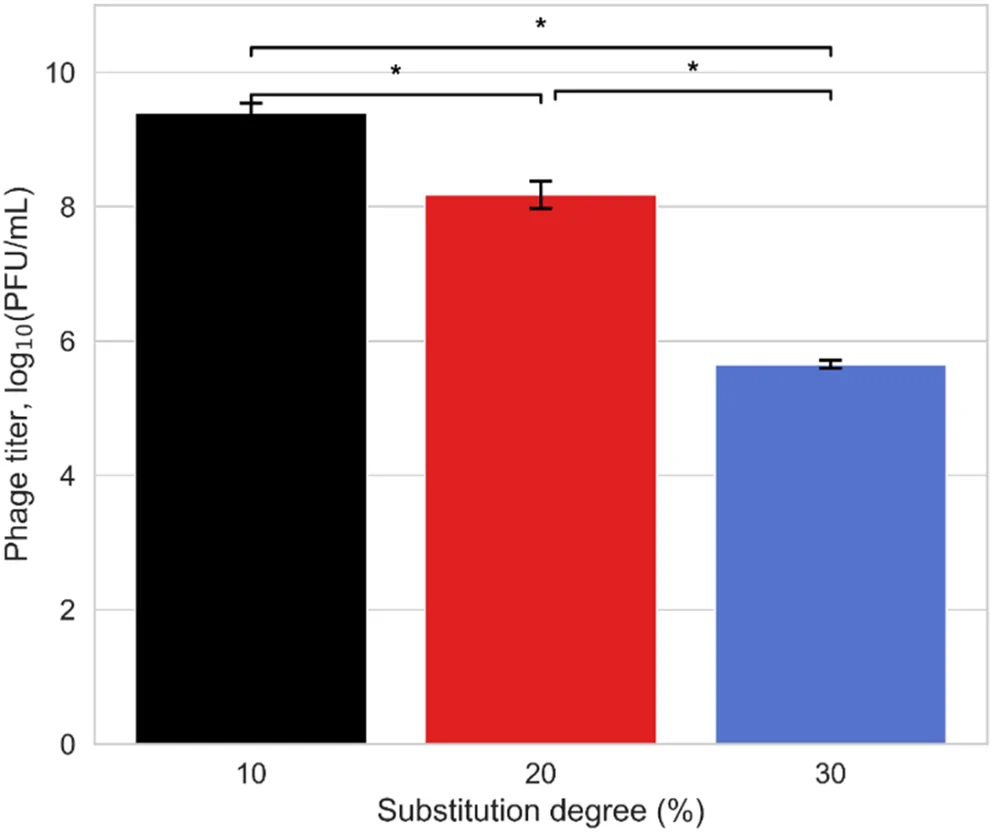
Phage titers of ϕ-OCS-OA nanoparticles with 10, 20, and 30% OA substitution degrees. Data are expressed as mean ± standard deviation (*n* = 3) and presented as log_10_(PFU/mL). Asterisks indicate significant differences among formulations according to one-way ANOVA followed by Tukey’s post hoc test (*p* < 0.05).

These results underscore the role of the OCS-OA hydrophobicity in controlling phage release. The 10 and 20% formulations likely form more relaxed micelle-like nanostructures that facilitate phage diffusion and the subsequent release. Conversely, the tighter packing induced by the 30% derivative appears to hinder the phage escape from the polymeric matrix, resulting in lower recovered infectious titers. Importantly, empty OCS-OA nanoparticles and unmodified OCS did not exhibit intrinsic lytic activity (Figure 4B), indicating that the antibacterial effect was associated with the encapsulated phages rather than the polymeric carrier.

## Conclusions

In this study, OCS-OA nanoparticles were successfully synthesized via EDAC/NHS-mediated amidation at three substitution degrees (10, 20, and 30%). The results demonstrate that OA grafting effectively transforms the OCS into an amphiphilic polymer capable of spontaneous self-assembly in aqueous media. Surface tension analysis supported the self-assembled nanostructures and showed that increasing the OA substitution degree decreased the CMC, consistent with stronger hydrophobic interactions.

The bacteriophage vB_Eco_K-02 was efficiently encapsulated within the OCS-OA nanoparticles (>90%), while preserving its biological viability. Physicochemical characterization revealed spherical nanostructures and demonstrated that colloidal stability strongly depends on the OA substitution degree, with the 10 and 20% formulations exhibiting superior stability over 13 days. However, beyond the colloidal stability of ϕ-OCS-OA nanoparticles under aqueous conditions, the stability and functional performance under inhalation-relevant conditions, including mucus environments, simulated fluid lung, and nebulization on particle integrity, were not evaluated in the present study. Therefore, these studies should be systematically investigated in future work to establish the suitability of this platform for pulmonary phage delivery.

Importantly, lytic activity assays against a clinical CF-associated *E. coli* strain demonstrated that 10 and 20% OCS-OA formulations preserved phage infectivity and enabled effective antibacterial activity. In contrast, the 30% formulation showed reduced efficacy, likely because of hindered phage diffusion from the polymeric matrix. Although the present study demonstrates that OCS-OA nanoparticles preserve the lytic activity of the encapsulated bacteriophage vB_Eco_K-02, complete phage release kinetics were not established. Because phages are large biological entities, conventional dialysis-based assays commonly used for low-molecular weight drugs are not directly applicable to phage release studies without prior validation, since membrane retention and adsorption of nanoparticle-associated phages may affect quantification. Therefore, the present findings provide evidence that phage infectivity and lytic activity are retained after encapsulation rather than as a definitive proof of controlled release. The long-term release behavior of infectious phage particles in relevant physiological media, such as simulated lung fluid, remains an important limitation of this study and should be addressed in future work.

Overall, OCS-OA nanoparticles represent a promising platform for bacteriophage encapsulation and delivery with potential application in the treatment of bacterial infections.

## Future Studies

OCS-OA nanoparticles showed high positive ζ-potential values, which may contribute to colloidal stability in aqueous media; this surface charge may also influence their interaction with the mucus found in the respiratory conducts. Positively charged nanoparticles can interact strongly with negatively charged mucus components,^18,19^ which, on the one hand, potentially increase mucoadhesion and local residence time^20,21^ but, on the other hand, limit diffusion throughout the mucus barrier. Therefore, future studies must evaluate the behavior of OCS-OA nanoparticles in simulated lung fluid, mucus microenvironment, or *ex vivo* sputum models to determine their diffusion capacity, phage infectivity, and antibacterial activity under airway-relevant conditions. Surface charge modulation may also be required to optimize the balance between colloidal stability, mucus interaction, and bacteriophage delivery.

## Supplementary Material



## Notes

Identification of bacterial strains; bacteriophage isolation; and interfacial parameters (CMC, Γ_max_, and *A*_min_) derived from surface tension (γ) measurements (PDF)

## References

[1] Heching M., Slomianksy L., Mussaffi H., Prais D., Weinberg J., Kramer M. R. (2024). Dual Inhaled Antibiotics for Treatment of Pulmonary
Exacerbations in Cystic Fibrosis-A Real Life Pilot Study. Respir. Med. Res..

[2] Burgel P.-R., Southern K. W., Addy C., Battezzati A., Berry C. A., Bouchara J.-P., Brokaar E. J., Brown A., Azevedo P., Durieu I., Ekkelenkamp M. B., Finlayson F., Forton J., Gardecki J., Hodkova P., Hong G., Lowdon J., Madge S., Martin C., McKone E. F. (2024). Standards for the Care of People with Cystic
Fibrosis (CF); Recognising and Addressing CF Health Issues. J. Cystic Fibrosis.

[3] Flume P. A., Mogayzel P. J., Robinson K. A., Goss C. H., Rosenblatt R. L., Kuhn R. J., Marshall B. C. (2009). Cystic Fibrosis
Pulmonary Guidelines. Am. J. Respir. Crit. Care
Med..

[4] Thornton C. S., Parkins M. D. (2023). Microbial Epidemiology of the Cystic
Fibrosis Airways:
Past, Present, and Future. Semin. Respir. Crit.
Care Med..

[5] Perikleous E. P., Gkentzi D., Bertzouanis A., Paraskakis E., Sovtic A., Fouzas S. (2023). Antibiotic Resistance in Patients
with Cystic Fibrosis: Past, Present, and Future. Antibiotics.

[6] Edwards B. D., Somayaji R., Greysson-Wong J., Izydorczyk C., Waddell B., Storey D. G., Rabin H. R., Surette M. G., Parkins M. D. (2020). Clinical Outcomes Associated with *Escherichia
coli* Infections in Adults with Cystic Fibrosis: A Cohort
Study. Open Forum Infect. Dis..

[7] Izydorczyk C., Waddell B., Edwards B. D., Greysson-Wong J., Surette M. G., Somayaji R., Rabin H. R., Conly J. M., Church D. L., Parkins M. D. (2020). Epidemiology of *E. coli* in Cystic Fibrosis Airways Demonstrates the Capacity
for Persistent
Infection but Not Patient-Patient Transmission. Front. Microbiol..

[8] Voronina O. L., Kunda M. S., Ryzhova N. N., Ermolova E. I., Goncharova E. R., Koroleva E. A., Kapotina L. N., Morgunova E. Y., Amelina E. L., Zigangirova N. A. (2025). Chronic *Escherichia coli* ST648 Infections in Patients with Cystic Fibrosis: The In Vitro
Effects of an Antivirulence Agent. Int. J. Mol.
Sci..

[9] Nichols D. P., Durmowicz A. G., Field A., Flume P. A., VanDevanter D. R., Mayer-Hamblett N. (2019). Developing Inhaled Antibiotics in Cystic Fibrosis:
Current Challenges and Opportunities. Ann. Am.
Thorac. Soc..

[10] Hitchcock N. M., Devequi Gomes Nunes D., Shiach J., Valeria Saraiva Hodel K., Dantas Viana
Barbosa J., Alencar Pereira Rodrigues L., Coler B. S., Botelho Pereira Soares M., Badaró R. (2023). Current Clinical
Landscape and Global Potential of Bacteriophage Therapy. Viruses.

[11] Cocorullo M., Stelitano G., Chiarelli L. R. (2024). Phage Therapy: An Alternative Approach
to Combating Multidrug-Resistant Bacterial Infections in Cystic Fibrosis. Int. J. Mol. Sci..

[12] Encinas-Basurto D., Martinez-Flores P. D., García J., Lopez-Mata M. A., García-González G., Rodea G. E., Eedara B. B., Mansour H. M., Juarez J. (2025). Latest Advances
in Inhalable Dry
Powder Bacteriophage Therapy for Pulmonary Infections. Pharmaceutics.

[13] Elsayed M. M., Elkenany R. M., EL-Khateeb A. Y., Nabil N. M., Tawakol M. M., Hassan H. M. (2024). Isolation and Encapsulation
of Bacteriophage with Chitosan
Nanoparticles for Biocontrol of Multidrug-Resistant Methicillin-Resistant *Staphylococcus aureus* Isolated from Broiler Poultry Farms. Sci. Rep..

[14] Saeedi M., Vahidi O., Moghbeli M. R., Ahmadi S., Asadnia M., Akhavan O., Seidi F., Rabiee M., Saeb M. R., Webster T. J., Varma R. S., Sharifi E., Zarrabi A., Rabiee N. (2022). Customizing Nano-Chitosan for Sustainable
Drug Delivery. J. Controlled Release.

[15] Luna M., Beltran O., Encinas-Basurto D. A., Ballesteros-Monrreal M. G., Topete A., Hassan N., López-Mata M. A., Reyes-Márquez V., Valdez M. A., Juarez J. (2022). High Antibacterial
Performance of Hydrophobic Chitosan-Based Nanoparticles Loaded with
Carvacrol. Colloids Surf., B.

[16] Rosen, M. J. ; Kunjappu, J. T. Surfactants and Interfacial Phenomena; John Wiley & Sons: New York, NY, 2012.

[17] García-González G., García-Pérez J. M., Martinez Flores P. D., Rivera Ríos R., Espinoza-Mellado M. D.
R., Velázquez-Guadarrama N., Encinas Basurto D. A., Juárez J., López Mata M. A., Eslava-Campos C. A., Rodea G. E. (2025). Isolation and Characterization of
Four New Coliphages against Extra Intestinal Pathogenic *Escherichia
coli* Isolates Recovered from Cystic Fibrosis Patients. ACS Omega.

[18] Jalal R. R., Ways T. M. M., Elella M. H. A., Hassan D. A., Khutoryanskiy V. V. (2023). Preparation
of Mucoadhesive Methacrylated Chitosan Nanoparticles for Delivery
of Ciprofloxacin. Int. J. Biol. Macromol..

[19] Abruzzo A., Giordani B., Miti A., Vitali B., Zuccheri G., Cerchiara T., Luppi B., Bigucci F. (2021). Mucoadhesive and Mucopenetrating
Chitosan Nanoparticles for Glycopeptide Antibiotic Administration. Int. J. Pharm..

[20] Suh J. W., Lee J.-S., Ko S., Lee H. G. (2016). Preparation
and
Characterization of Mucoadhesive Buccal Nanoparticles Using Chitosan
and Dextran Sulfate. J. Agric. Food Chem..

[21] Silva M. M., Calado R., Marto J., Bettencourt A., Almeida A. J., Gonçalves L. M.
D. (2017). Chitosan Nanoparticles
as a Mucoadhesive Drug Delivery System for Ocular Administration. Mar. Drugs.

