# Supplementary material for: Oleic Acid-Modified Oligochitosan Nanoparticles Preserve Bacteriophage Lytic Activity against Clinical Escherichia coli Isolates from Patients with Cystic Fibrosis
Source: ACS Omega. 2026 Sep 8;11(39):59113–9. doi: 10.1021/acsomega.6c04685 (PMC13647333; doi:10.1021/acsomega.6c04685)
Supplement: Supplementary file 1 [file ao6c04685_si_001.pdf]

Oleic Acid–Modified Oligochitosan Nanoparticles Preserve Bacteriophage Lytic Activity Against Clinical *Escherichia coli* Isolates from Patients with Cystic Fibrosis

Joselyn Aime García-Mar[1], *Patricia Dolores Martínez-Flores*[1], *David Encinas-Basurto*[1], *Gerardo García-González*[2], *Gerardo E. Rodea-Montealegre*[3], *Refugio Pérez-González*[4], *Viviana Reyes-Márquez5*, *Marco Antonio López-Mata*[6], *Natalia Hassan*[7], *Antonio Topete*[8], *Josué Juárez*[1*]

josue.juarez@unison.mx*

1. Departamento de Física, Facultad de Ciencias Exactas y Naturales, Universidad de Sonora, Hermosillo CP 83000, México.
2. Departamento de Microbiología, Facultad de Medicina y Hospital Universitario "Dr. José Eleuterio González", Universidad Autónoma de Nuevo León, Monterrey 64460, México.
3. Laboratorio de Investigación en Microbiología y Resistencia Bacteriana, Hospital infantil de México, Federico Gómez, Ciudad de México, 06720, Mexico.
4. Departamento de Investigación en Polímeros y Materiales, Universidad de Sonora, Hermosillo CP 83000, México.
5. Departamento de Ciencias Químico-Biológicas, Facultad Interdisciplinaria de Ciencias Biológicas y de Salud, Universidad de Sonora, Hermosillo CP 83000, México.
6. Departamento de Ciencias de la Salud, Facultad Interdisciplinaria de Ciencias Biológicas y de Salud, Universidad de Sonora, Cajeme CP 85010, México.
7. Programa Institucional de Fomento a la Investigación, Desarrollo e Innovación, Universidad Tecnológica Metropolitana, Santiago, Chile.
8. Departamento de Física de Partículas, Instituto de Materiales (iMATUS) e Instituto de Investigación Sanitaria (IDIS), Universidad de Santiago de Compostela, 15782, España.

Identification of Bacterial Strains

Wastewater samples were collected, and particulate matter was removed by centrifugation (10,000 rpm, 10 mins, 4°C) followed by filtration through a 0.22 μm PES syringe filter to separate viral particles from bacteria and other microorganisms. Multiple clinical strains of *Escherichia coli* were screened as potential hosts for the viral particles present in the filtered samples. Each bacterial strain was tested using the double-layer agar assay with Biotryptase broth and bacteriological agar as culture media. Samples showing plaque formation (lysis zone) were selected, and the corresponding phages were isolated and stored at 4°C for further analysis.

Bacteriophage Isolation

Selected *E. coli* host strains were grown overnight at 37 °C with constant agitation. The following day, bacteriological agar was melted and maintained at 50 °C in a water bath. Soft agar overlays were prepared by inoculating 10 μL of each bacterial culture into molten soft agar, followed by the addition of 90 μL of the corresponding phage-containing suspension. 10-fold serial dilutions of each phage suspension were prepared and plated to generate the top layer. After solidification, plates were incubated overnight at 37 °C.

Once plaque formation was confirmed, 5 μL of DNase and RNase were added and incubated for 30 min. Then, NaCl was added to a final concentration of 0.5 M to facilitate removal of bacterial nucleic acid contaminants, followed by incubation for 1 hour at 4 °C. The samples were centrifuged (8,000 rpm, 10 min, Velocity 14R, Dynamica Scientific), and polyethylene glycol (PEG) was added to the supernatant to precipitate the phages overnight at 4°C. The phage pellets were collected by centrifugation (8,000 rpm, 10 min, 4 °C) and resuspended in 1 mL of TM buffer. Chloroform was then added to promote phase separation, and the suspension was centrifuged

(14,000 rpm, 10 min, 4 °C). The aqueous phase containing the purified phages was recovered. Finally, plaque assays were performed using serial dilutions to quantify plaque forming units per milliliter (PFU/mL).

Table S1

Interfacial parameters (CMC, Γ_{max}, and A_{min}) derived from γ measurements.

OCS-OA	CMC (g/mL)	Γ_{max} (mol/m^2)	A_{min} (nm^2)
10%	4×10^{-4}	1.65	2.01
20%	1.9×10^{-4}	3.39	0.99
30%	2.6×10^{-5}	5.3	0.62
